# Detecting predictive androgen receptor modifications in circulating prostate cancer cells

**DOI:** 10.18632/oncotarget.3925

**Published:** 2015-04-23

**Authors:** Julie Steinestel, Manuel Luedeke, Annette Arndt, Thomas J. Schnoeller, Jochen K. Lennerz, Carina Wurm, Christiane Maier, Marcus V. Cronauer, Konrad Steinestel, Andres J. Schrader

**Affiliations:** ^1^ Clinic of Urology, University Hospital Ulm, Ulm, Germany; ^2^ Clinic of Urology, University Hospital Muenster, Muenster, Germany; ^3^ Institute of Pathology and Molecular Pathology, Bundeswehrkrankenhaus Ulm, Ulm, Germany; ^4^ Massachusetts General Hospital/Harvard Medical School, Boston, MA, USA; ^5^ Gerhard-Domagk Institute of Pathology, University of Muenster, Muenster, Germany

**Keywords:** castration-resistant prostate cancer, androgen receptor modification, splice variants, circulating tumor cells

## Abstract

Molecular modifications of the androgen receptor (AR) can cause resistance to androgen deprivation therapy (ADT) in prostate cancer patients. Since lack of representative tumor samples hinders therapy adjustments according to emerging AR-modifications, we evaluated simultaneous detection of the two most common AR modifications (AR-V7 splice variant and *AR* point mutations) in circulating tumor cells (CTCs). We devised a single-tube assay to detect AR-V7 splice variants and *AR* point mutations in CTCs using immunomagnetic cell isolation, followed by quantitative real-time PCR and DNA pyrosequencing. We prospectively investigated 47 patients with PSA progression awaiting therapy switch. Comparison of response to newly administered therapy and CTC-AR-status allowed effect size estimation. Nineteen (51%) of 37 patients with detectable CTCs carried AR-modifications. Seventeen patients carried the AR-V7 splice variant, one harbored a p.T878A point mutation and one harbored both AR-V7 and a p.H875Y mutation. We estimated a positive predictive value for response and non-response to therapy by AR status in CTCs of ~94%. Based on a conservative calculation, we estimated the effect size for molecularly-informed therapy switches for prospective clinical trial planning to ~27%. In summary, the ability to determine key resistance-mediating AR modifications in CTCs has the potential to considerably improve prostate cancer treatment.

## INTRODUCTION

Therapy of advanced prostate cancer is based on interference with androgen receptor (AR) signaling and androgen deprivation therapy (ADT) has been firmly established as the principal therapeutic approach [1]. Unfortunately, all patients ultimately develop resistance to primary ADT (surgical/medical castration) as well as novel hormonal therapies (next-generation ADT), which either suppress the synthesis of extragonadal androgens (e.g. abiraterone) or target the androgen receptor directly (e.g. enzalutamide).

Resistance to both primary and next-generation ADT is frequently caused by molecular AR-modifications. First, the most common AR-modification is an AR splice variant (AR-V7) that renders cancer cells resistant to otherwise effective therapy (e.g. abiraterone and enzalutamide) [2]. For patients with AR-V7-positive tumors, alternative therapies are currently being evaluated [3]. Second, the less frequent point mutations of the *AR* gene carry clinical importance due to their specific functional differences (Table 1) [4-7]. In sum, knowledge of the given AR-modification status would help to select the most effective therapy for each individual patient. However, assessment of the AR-modification status would require representative tumor samples at each time of PSA progression, which is impractical for therapeutic monitoring. Consequently, assessment of individual AR modifications is currently not part of the therapeutic decision tree and therapy switches occur uninformed with respect to the AR-modification status. 

**Figure 1:**
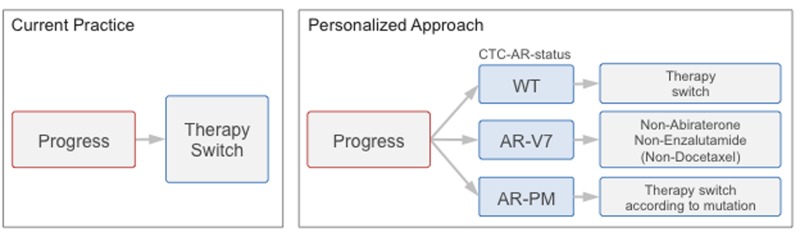
Study rationale. Current practice: Therapy switch at progress (PSA progress or progressive disease) occurs molecularly uninformed. Personalized approach: Evaluation of androgen receptor status in circulating tumor cells (CTC-AR-status) at time of progression enables matching of therapy to the individual resistance profile.

Circulating tumor cells (CTC) hold the promise to provide easy access for cancer characterization. The number of CTCs has been demonstrated as an indicator of disease aggressiveness and tumor burden and Miyamoto et al., for example, determined AR signaling status in patients under ADT as a possible indicator for therapy success [8] [9]. CTCs may thus provide easy access to the patient’s current AR status and thereby indicate the resistance and sensitivity profile of the tumor. Following these notions, Antonarakis et al. recently reported the detection of AR-V7 mRNA in CTCs as indicative of resistance to both abiraterone and enzalutamide [10]. The group did, however, not assess point mutations of the *AR* gene in CTCs, while there is an implicit understanding that treatment decisions based on both AR-splice variants and point mutations would significantly improve therapy response rates. This point formed a rationale for our study, and we propose that treatment decisions based on CTC assessment should consider both AR-modifications (Figure 1). 

Here, we evaluated whether it is possible to simultaneously detect the two most common AR modifications - the AR-V7 splice variant and *AR* hotspot mutations - in CTCs from a single blood sample. Beside demonstration of general feasibility, we assess predictive properties with respect to subsequent therapies, which collectively underscore the potential of liquid biopsies in the treatment of advanced prostate cancer.

## RESULTS

### *In vitro* validation

A prerequisite for a clinically valid assay in this setting is the ability to reliably detect and characterize prostate cancers cells in peripheral blood. First, we demonstrated absence of qPCR products for prostate-specific mRNAs (KLK3, AR) in blood from a healthy male and female donor. In spiking experiments, using various dilutions of prostate cancer cells in blood from the same donors, we successfully detected prostate-specific mRNAs, the AR-V7 splice variant, as well as the c.2632A > G; p.T878A mutation in LNCaP-spiked blood (see Supplementary Methods). These findings provide proof-of-principle that our method allows simultaneous detection of AR-V7 and *AR *point mutations.

**Figure 2:**
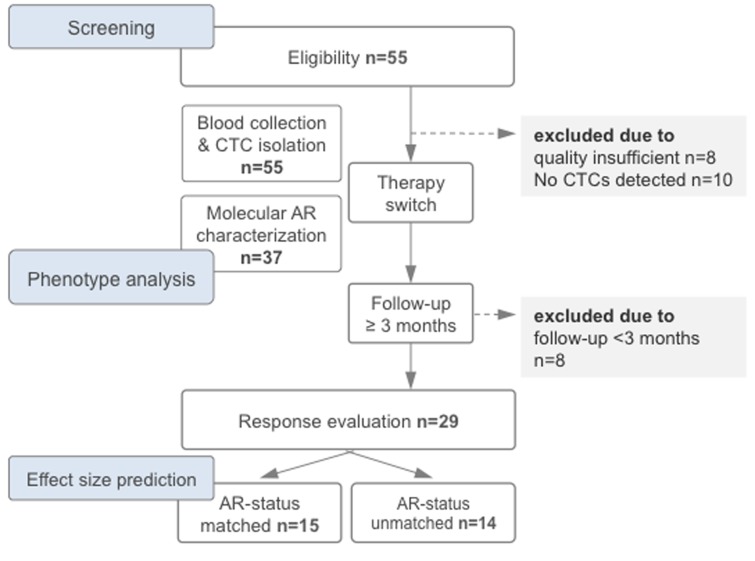
Study flow chart depicting the timeline of therapy switch, blood draw, circulating tumor cell (CTC) analysis and evaluation of response rates. For estimation of effect size according to molecularly matched and unmatched therapy switches see results. Abbreviation: AR, androgen receptor gene (here assessed in CTCs).

### CTCs in the study cohort

**Figure 3:**
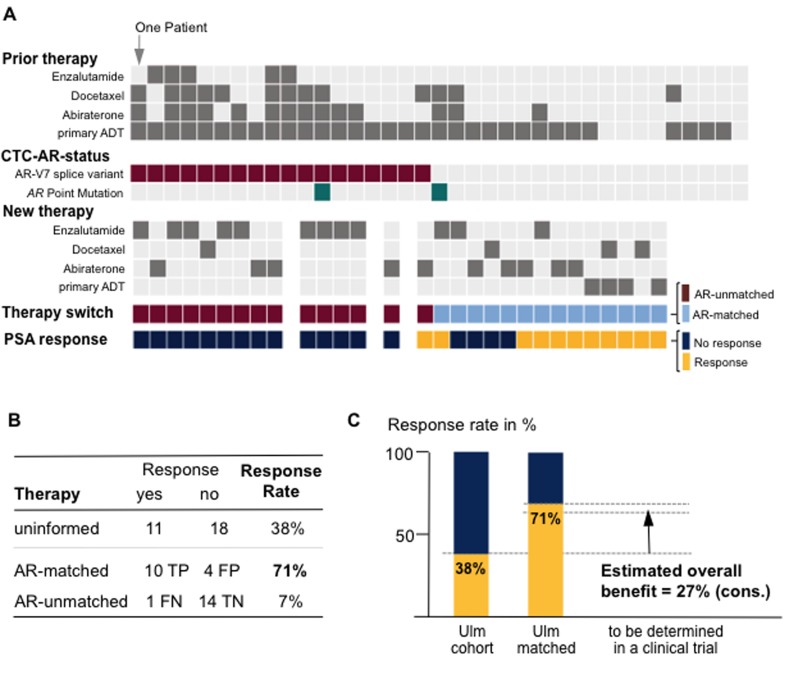
Study results. **A.** Overview of prior and new therapies along with the androgen receptor status in the circulating tumor cells (CTC-AR-status) for each study patient. Therapy switch in our study occurred molecularly uninformed (see Figure 2); however, comparison of newly administered therapy and CTC-AR-status allowed assignment as molecularly AR-matched *vs*. AR-unmatched. We defined ‘response’ as PSA reduction ≥50%. Abbreviation: ADT, androgen-deprivation therapy. **B.** Comparison of response rates between uninformed and molecularly/AR-status matched *vs*. unmatched. Abbreviations: TP, true positive; FP, false positive; FN, false negative; TN, true negative. **C.** Effect size estimation for planning of a molecularly stratified, controlled clinical trial. Note, we account for the 7% response rate in the AR-unmatched subgroup.

Clinico-pathological data of the 47 patients that fulfilled enrollment criteria are summarized in Table 2 (all patients). We detected CTCs in peripheral blood from 37 patients (79% of the cohort). These patients had significantly higher concurrent PSA levels (*p* = 0.03) with one notable exception. Briefly, in this patient with undifferentiated metastasized prostate carcinoma, we detected CTCs although the PSA level was 0.1ng/ml, underscoring the assumption that PSA level alone does not always accurately reflect tumor burden [11]. Hallmarks of tumor aggressiveness at the time of diagnosis (initial PSA or Gleason score) did not correlate with detectability of CTCs (*p*-range = 0.69-0.72). Figure 3A provides an overview of previous prostate cancer therapy, AR-status and subsequent therapy for all CTC-positive patients.

### Clinico-pathological correlation of AR-modifications in CTCs

AR-V7 qPCR revealed splice variants in 18 of 37 CTC-positive patients (49%). Presence of AR-V7 correlated significantly with metastatic disease (*p *= 0.046), but not with other parameters classically associated with aggressive clinical course (i.e., initial PSA or Gleason score; *p*-range: 0.28-0.74; Table 3). Presence of AR-V7 showed significant associations with prior primary ADT alone (*p* = 0.046), previous treatment with abiraterone (*p* = 0.007), enzalutamide (*p* = 0.02), or docetaxel (*p* = 0.02), as well as with the number of prior therapies (*p* = 0.004). Specifically, examining the percentage of patients harboring the AR-V7 in relation to prior therapy combinations showed the following results: 28.6% primary ADT alone, 66.7% primary ADT + docetaxel, 80% primary ADT + next-generation ADT (abiraterone and/or enzalutamide), and 80% primary ADT + next-generation ADT + docetaxel. The significantly higher rate of AR-V7 in patients with multiple prior therapies (*p* = 0.005), and our finding that no therapy-naïve patient was AR-V7-positive argues for acquisition of the splice variant during therapy.

*AR *sequencing in 37 patients revealed 2 *AR* point mutations in 2 different patients. In one patient we found a c.2632A > G, p.T878A mutation with a mutant-to-wildtype ratio of 0.92 (Supplementary Figure 1A) [12]. The p.T878A mutation is an acknowledged cause of resistance to abiraterone (Table 1) which is in accordance with the patient’s non-response to prior abiraterone treatment. In another patient, we detected a c.2623C > T, p.H875Y point mutation with an allelic ratio of 0.11 (Supplementary Figure 1B). Interestingly, both patients had previously been treated with abiraterone and docetaxel. In the first patient the p.T878A mutation was detected in the absence of the AR-V7 splice variant, whereas the second patient harbored both AR-V7 and p.H875Y. Based on these findings, we tested the predictive properties of our assay.

### AR-modifications in CTCs as a prediction tool for therapeutic response

During follow-up after therapy switch, we observed a biochemical response in 71% of AR-V7 negative patients (*n* = 10/14) whereas only one AR-V7 positive patient (*n* = 1/15, 7%) responded to the following therapy. While these differences reached statistical significance (*p* < 0.001), the response characteristics of subgroups by treatment type were not as clear. For example, two of three AR-V7-negative patients who received docetaxel responded, while the AR-V7 positive patient had no benefit from docetaxel treatment. With respect to follow-up in the p.T878A-positive patient, we observed a response to subsequent treatment with enzalutamide (PSA decline from 32 to 16ng/ml). In contrast, the p.H875Y/AR-V7-positive patient, showed no biochemical response to subsequent enzalutamide treatment.

To determine predictive properties of our assay in patients with outcome information (*n* = 29), we dichotomized therapy outcome into either response or non-response. We defined response as reduction of serum PSA to < 50% maintained for ≥4 weeks at any time after the initiation of therapy. When we assumed that assessment of the AR modification status predicts response as well as non-response, the ‘positive’ predictive value would add up to ~94%. 

In our study we switched therapies molecularly uninformed and observed a response rate of 38% (*n* = 11/29). When splitting up patients into subgroups with molecularly matched (*n* = 14) and –unmatched therapy switches (*n* = 15), we observed 71.4% and 6.6% response rates, respectively (Figure 3B for details). We calculated the conservative estimated overall benefit (see methods) of a molecularly informed treatment decision to: ~27% (Figure 3C). 

**Table 1:** Selected Resistance Mechanisms by Type of Androgen Receptor Gene (AR) Modification.

Modification	Resistance to	Mechanism	Reference
**Alternative splicing**			
AR-V567 AR-V7	General ADT General ADT	Constitutive activation Constitutive activation	[26] [10, 16]
**AR point mutations**			
p.W742C p.W742L p.E873Q p.H875Y p.F877L p.T878A p.T878S p.D880G	Bicalutamide Bicalutamide Abiraterone, Cyproterone acetate Abiraterone, Nilutamide, Hydroxyflutamide Enzalutamide Abiraterone, Flutamide, Hydroxyflutamide Flutamide, Abiraterone Bicalutamide	Antagonist-to-agonist switch Antagonist-to-agonist switch Receptor promiscuity Receptor promiscuity, antagonist-to-agonist switch Antagonist-to-agonist switch Receptor promiscuity, antagonist-to-agonist switch Antagonist-to-agonist switch, receptor promiscuity Antagonist-to-agonist switch (controversial)	[27] [28] [6] [29] [30] [31] [22, 29] [19]

Abbreviations: ADT, androgen deprivation therapy; AR-V567, AR-V7, androgen-receptor splice variant messenger RNAs.

**Table 2:** Demographic and Clinical Characteristics in Prostate Cancer Patients Screened for CTC.

Characteristics	All Patients N = 47
Detection of CTCs yes no	n = 47 37 10
Age in years Median (range)	n = 47 75 (53-87)
Time since diagnosis in years Median (range)	n = 47 5 (1-16)
PSA level at time of blood draw Median (range)	n = 47 96.5 ng/ml (0.1-4282)
PSAi level Median (range)	n = 39 27.9 ng/ml (0.6-5000)
Gleason Score ≤ 7 ≥ 8	n = 46 19 27
Prior use of primary ADT yes no	n = 47 41 6
Prior use of abiraterone yes no	n = 47 18 29
Prior use of docetaxel yes no	n = 47 17 30
Prior use of enzalutamide yes no	n = 47 5 42
Metastases yes no	n = 47 40 7
Lymph node metastases yes no	n = 47 24 23
Bone metastasis yes no	n = 47 39 8
Visceral metastasis yes no	n = 47 8 39
PSA responsea to abiraterone treatment yes no	n = 14 6 8
PSA response to enzalutamide treatment yes no	n = 12 2 10
PSA response to enzalutamide or abiraterone treatment yes no	n = 26 8 18
PSA response to any subsequent treatmentb yes no	n = 34 13 21

a to subsequent treatment; b including docetaxel.

**Table 3:** Demographic and Clinical Characteritistics of AR-V7 Genotype-Specific Subsets of Patients with Advances Prostate Cancer.

Characteristics	AR-V7 Negative N = 19	AR-V7 Positive N = 18	P-value*
Age in years Median (range)	n = 19 72/56-87	n = 18 74 (53-82)	0.69
Time since diagnosis in years Median (range)	n = 19 1(1-13)	18 7(1-16)	0.216
PSA level at time of blood draw Median (range)	n = 19 88.6 (0.1-1374)	n = 18 239.9 (13.9-4282)	0.274
PSAi level Median (range)	n = 17 30.0 (0.6-1020)	n = 14 16.1 (5-906)	0.284
Gleason Score ≤ 7 ≥ 8	n = 19 8 11	n = 17 6 11	0.742
Prior use of primary ADT yes no	n = 19 14 5	n = 18 18 0	0.046*
Prior use of abiraterone yes no	n = 19 3 16	n = 18 11 7	0.007*
Prior use of docetaxel yes no	n = 19 3 16	n = 18 8 10	0.017*
Prior use of enzalutamide yes no	n = 19 0 19	n = 18 5 13	0.02*
Metastases yes no	n = 19 14 5	n = 18 18 0	0.046*
Lymph node metastases yes no	n = 19 9 10	n = 18 11 7	0.515
Bone metastasis yes no	n = 19 14 5	n = 18 18 0	0.046*
Visceral metastasis yes no	n = 19 4 15	n = 18 3 15	1.0
PSA responsea to abiraterone treatment yes no	n = 5 3 2	n = 5 1 4	0.524
PSA response to enzalutamide treatment yes no	n = 3 2 1	n = 9 0 9	0.045*
PSA response to enzalutamide or abiraterone treatment yes no	n = 8 5 3	n = 14 1 13	0.011*
PSA response to any subsequent treatmentb yes no	n = 14 10 4	n = 15 1 14	<0.001*

a to subsequent treatment; b including docetaxel

P-values from Fisher’s exact test for dichotomous variables and student’s t-test for continuous variables

## DISCUSSION

The present study demonstrates for the first time the feasibility to detect the two main AR modifications in CTCs of patients with advanced prostate cancer in a single blood tube. Since we show that an informed personalized treatment decision has the potential to significantly reduce the rate of non responders, CTC- based molecular testing can prevent patients from receiving inefficient therapy.

Parallel detection of AR-V7 and *AR* hotspot mutations in CTCs has not been previously reported. While there are several studies demonstrating isolation, enumeration, imaging, and molecular-genetic characterization of CTCs [9, 13, 14], parallel detection of the two main AR modifications in CTCs is lacking. Recently, Antonarakis et al. [10] reported detection of AR-V7 splice variant in circulating prostate cancer cells. While the fraction of patients with CTCs harboring AR-V7 was compatible (~50% vs. here 49%), Antonarakis et al. did not determine clinically relevant *AR* point mutations in CTCs. Our approach now combines a commercially available, immunoprecipitation-based method for CTC isolation with quantitative real-time PCR analysis for the detection of AR-V7 and subsequent DNA pyrosequencing of *AR *mutation hotspots. We demonstrate overall feasibility and provide a straightforward method for parallel detection of the two main AR modifications in CTCs as a single-tube assay. 

The estimated overall benefit from molecularly informed treatment decisions is the central pivot point of our study. There is an implicit understanding supported by recent studies [10, 12] that there is a benefit from molecular characterization of AR in CTCs in prostate cancer. The effect size will have to be determined in randomized controlled prospective clinical trials. Here, we used the interventional (yet retrospective) design of our study (Figure 2) to estimate this overall benefit. Therefore, we performed a statistical calculation and applied stringent criteria working under the statistical premise to assume the most pessimistic situation [15]. Specifically, we started with the response rate of the AR-matched subgroup (71%) and subtracted the response rate observed when treatment decisions are made molecularly uninformed (as in our study). Note, that this subtraction of 38% already incorporates all incorrectly predicted patients (Figure 3B; *n* = 4 false positive + *n* = 1 false negative). Despite the small number of patients, we noted a measurable benefit of a molecularly unmatched therapy (in our cohort 1 patient = 7%; Figure 3B, false negative). We decided to additionally subtract this “false-negative” value again, as it statistically argues against molecularly matched therapy and derives the most pessimistic assumption. Clearly, we are aware that this estimation is an imperfect estimate of the overall benefit and that the true effect size will have to be determined. Nonetheless, for future trial design or statistical predictions, we provide a conservative estimate of an overall benefit of 27%.

Correlating AR modification status with treatment response, we made three key findings. First, we noted that parameters of tumor aggressiveness at the time of diagnosis (i.e., initial PSA level or Gleason score) did not correlate with the presence of AR-V7. On the other hand, presence of AR-V7 was associated with the number of prior therapies. Together we interpret these associations as evidence for development of AR-V7 under therapy. This interpretation is supported by recently published *in vitro* data where treatment of two prostate cancer cell lines with either abiraterone or enzalutamide increased the expression of constitutively active AR-Vs (AR-V7 and AR-V567es) [16]. Furthermore, the emergence of AR-V7 under therapy together with the fact that 93% of AR-V7-positive patients in our study showed no PSA response to subsequent therapy may explain the limited efficacy of sequential therapies (enzalutamide–abiraterone or abiraterone-enzalutamide) [17]. However, one of our AR-V7-positive patients responded to abiraterone treatment with a PSA decrease. This finding is of considerable clinical interest as it indicates that some patients may in fact benefit from secondary ADT despite the presence of AR-V7, which is inconsistent with recently published data [10].

The second key finding results from analysis of the two patients with *AR* mutations. Specifically, both patients with *AR* point mutations were pre-treated with abiraterone. This observation supports recent reports about the emergence of these mutations under abiraterone therapy [18], where point mutations mediate additional responsiveness to oestrogens, progestins, cyproterone acetate and hydroxyflutamide [19-22]. Notably, patients differed in response to therapy switch to enzalutamide. Specifically, while the p.T878A-positive patient showed a PSA response, the patient with CTCs harboring both the p.H875Y mutation and the AR-V7 splice variant showed no response to subsequent enzalutamide treatment. Based on a previous report, showing presence of AR-V7 to be sufficient for resistance to enzalutamide in p.H875Y-mutant 22Rv1-cells *in vitro *[23], we interpret the non-response as related to the coexistence of the AR-V7 splice variant. Moreover, the low allelic ratio of the p.H875Y mutation (0.11) might be a second clue for the coexistence of two different CTC clones with individual AR-modifications in this patient, possibly reflecting biological heterogeneity of the primary tumor [24]. 

The third key finding is related to the observation that two of the three AR-V7-negative patients who received docetaxel responded to therapy. While the number of examined patients precluded formal statistical testing of response rates of AR-V7-positive vs. AR-V7-negative patients to docetaxel, one docetaxel-treated patient harbored the AR-V7 splice variant and showed no therapy response. In light of recent findings indicating that AR-V7 expression may also constitute a mechanism of resistance to taxanes [25], a more detailed analysis of this patient subgroup will be of significant interest. 

The small number of patients included in this first report has to be acknowledged as a clear limitation to the study; moreover, the predictive value of the assay is limited to patients with detectable CTCs. Finally, focusing on AR hotspot regions might lead to a miss of other mutations with possible clinical implications. From our point of view, since CTCs were detectable in 79% of patients, a pragmatic and cost-effective approach focusing on well-documented AR-modifications holds great clinical promise, all the more since an overall benefit of 27% could already be demonstrated in this small sample size.

In summary, our results demonstrate the ability to reliably characterize the two main AR-modifications in CTCs of patients with advanced prostate cancer in a single-tube assay. This finding lays the foundations for a liquid biopsy-based, personalized treatment decision that can prevent patients from receiving inefficient systemic therapy.

## MATERIALS AND METHODS

### Study design

We designed a prospective observational study (Figure 2) and obtained approval from the institutional review board at Ulm University (ethic vote no. 08/2014). All patients provided written informed consent.

### Patient selection

We prospectively enrolled patients with advanced prostate cancer who underwent treatment at the university medical center Ulm. To compose the study cohort, we applied the following inclusion criteria: histologically confirmed prostatic adenocarcinoma, progressive disease, and signed consent form. We defined progressive disease as PSA progression and/or radiographic progression (according to RECIST 1.1) irrespective of the stage of disease. PSA-Progression was defined as ≥50% decline in PSA from baseline maintained for ≥4 weeks at any time after the initiation of therapy. We enrolled patients irrespective of type, line, or sequence of prior therapies; however, therapy switch to any of the approved anticancer drugs had to occur after blood draw. Inclusion in other prospective clinical trials was allowed. For each enrolled patient we obtained epidemiological, clinical, and tumor-specific data from the medical record. Study start was February 2014 and we followed patients until October 2014.

### Validation experiments

To determine limits of detection for CTC isolation from peripheral blood, we performed spiking experiments with defined numbers of cultured prostate cancer cells (LNCaP). To determine detection limits (and baseline signals) of *AR *point mutations in pyrosequencing experiments, we used undiluted or admixed cDNA from prostate cancer cell lines harboring either wildtype sequence (VCaP) or different *AR* point mutations (LNCaP, 22Rv1). 

### Isolation of CTCs from peripheral blood

We collected 7.5 ml of peripheral venous blood in EDTA collection tubes and stored samples immediately at 4°C. CTC isolation procedure started within 1-24 hours after blood draw. For CTC isolation, we employed the AdnaTest ProstateCancerSelect Kit (AdnaGen, Langenhagen, Germany). Initial tests revealed that washing steps with PBS+0.05% bovine serum albumine reduced the loss of magnetic beads to plastic surfaces. Otherwise we followed the instructions provided by the manufacturer. 

*Isolation of RNA from CTCs* employed the AdnaGen ProstateCancerDetect Kit (AdnaGen, Langenhagen, Germany). 

### Quantitative real-time PCR and AR-V7 detection

To achieve mRNA specificity (and avoid false positive signals from residual DNA), we designed qPCR primers that span exon-exon boundaries. We assessed mRNA levels of AR-V7, KLK3 (PSA), ALAS1, G6PD, and AR exons 5 and 8 with the QuantiFast Multiplex RT-PCR+R Kit (Qiagen, Hilden, Germany) on a ViiA™ 7 Real-Time PCR system (Life Technologies, Carlsbad, USA). To rule out contamination, we analyzed patient samples along with their corresponding reverse transcription and qPCR non-templates. To determine the relative AR-V7 expression levels, we applied the ΔCt method (*AR* Exon 8 expression level served as internal reference). 

### AR mutation analysis

To allow simultaneous mutation analysis, we designed the qPCR products of AR exons 5 and 8 to specifically cover known AR hotspot point mutations (Table 1). Specifically, using separate forward and reverse reactions, we generated pPCR products with one biotinylated and one opposing unmodified primer. Biotin-streptavidin based strand separation allowed subsequent bidirectional pyrosequencing. We immobilized the biotinylated qPCR products using streptavidin sepharose beads followed by strand separation on the PyroMark Q24 Vacuum Workstation (Qiagen, Hilden, Germany) and performed annealing of the appropriate sequencing primers at 80 °C for 2 min followed by two consecutive cooling steps at room temperature for 2 and 15 min, respectively. Sequencing and analysis employed the PyroMark Q24 and software version 2.0 (Qiagen, Hilden, Germany).

Primer sequences and further experimental details are given in the Supplementary Material section.

### Statistics and estimation of effect size

We assessed correlations between clinico-pathological parameters (Tables 2 and 3) using the Fisher’s exact- or Mann-Whitney-U-test, as appropriate. For evaluation of therapeutic response we defined biochemical response as a larger than 50% reduction in serum PSA during treatment. Given that the molecular status can predict response as well as non-response, we defined the overall performance of our test as the fraction of correctly predicted responses and non-responses in our cohort and calculated the positive predictive value. Since therapy switch in our study occurred molecularly uninformed (Figure 2) we were able to retrospectively categorize newly administered therapy and CTC-AR-status as “molecularly AR-matched” vs. “AR-unmatched” (Figure 3A). The overall increase in response rate can be calculated as AR-matched response rate minus uninformed response rate. To provide a conservative estimate of the overall benefit, we took the incorrect predicted response rate in the AR-unmatched group into account. The resulting formula is: Estimated overall benefit = response rate-AR-matched – (response rate-uninformed + response rate-AR-unmatched). We used SPSS for statistical calculations (Version 21, IBM, Ehningen, Germany), the R-package for generation of heatmaps (www.r-project.org), and defined a two-sided *p* < 0.05 as statistically significant. 

## SUPPLEMENTARY MATERIALS FIGURE AND TABLES


