# Supplementary material for: Detecting predictive androgen receptor modifications in circulating prostate cancer cells
Source: Oncotarget. 2015 Apr 23;10(41):4213–23. doi: 10.18632/oncotarget.3925 (PMC6609250; doi:10.18632/oncotarget.3925)
Supplement: Supplementary file 1 [file oncotarget-10-4213-s001.pdf]

Detecting predictive androgen receptor modifications in circulating prostate cancer cells

Supplementary material

		page
A.	Control experiments	2
B.	Quantitative real-time PCR	3
C.	DNA pyrosequencing	3
D.	Table 1: Primer and probe sequences	4
	Supp. Fig. 1: cDNA pyrosequencing of AR hotspots	5
	Supp. Fig. 2: Correlation of AR-V7/ARwt with max. PSA-response	6

A. Control experiments

Cell lines and cell culture conditions

We obtained VCaP, LNCaP and 22Rv1 cells from ATCC (Wesel, Germany) where they had undergone STR profiling for authentication purposes. Cells were kept under sterile cell culture conditions (37°C, 5% CO_2) and passaged at 70% confluence. We maintained VCaP cell line in DMEM medium (Gibco, Life Technologies, Carlsbad, USA) and LNCaP and 22Rv1 cell lines in RPMI 1640 medium (Gibco, Life Technologies, Carlsbad, USA). Both media were supplemented with 10% fetal calf serum (BioWest, Nuaille, France), 100U Penicillin and 100µg Streptomycin per ml (Pan Biotech, Aidenbach, Germany).

RNA isolation and Reverse Transcription

For total RNA isolation from approximately 2.5m VCaP, LNCaP and 22Rv1 cells, we used the RNeasy Plus Mini Kit (Qiagen, Hilden, Germany) as recommended by the manufacturer. Fourty ng RNA served as template for cDNA synthesis, performed with the Sensiscript Reverse Transcription Kit (Qiagen, Hilden, Germany). We used Oligo dT(23) and random nonamers (both from Sigma-Aldrich, Hamburg, Germany) in a final concentration of 0.5µl and 5µM, respectively. Recombinant RNasin Ribonuclease Inhibitor was obtained from Promega, Fitchburg, USA. Cycling was conducted in a Labcycler 96 Gradient (Sensoquest, Göttingen, Germany). For later use as positive control in qPCR measurements, we stored the final cDNA at -80°C.

Determination of background noise and sensitivity of the AR qPCR pyrosequencing

We performed multiple repeats of *AR*-wildtype VCaP cDNA pyrosequencing to determine the background noise level. The resulting data (peak heights) served as threshold for the following experiments, facilitating the reliable detection of AR mutations in low abundant CTC subclones. For the determination of pyrosequencing sensitivity, we took advantage from the fact that the cell lines LNCaP and 22Rv1 harbor the *AR* p.T878A and p.H875Y mutation, respectively. In VCaP, LNCaP and 22Rv1 cell lines, we quantified the relative expression levels of the *AR* gene and mixed accurate dilutions of cDNA templates to obtain a gradual increase in the respective mutation frequency between 0 and 100% in the final template. By this approach, we were able to identify the mutated to wildtype AR cDNA ratio at which the mutated sequence can be observed above the background noise level. Subsequent qPCR and pyrosequencing of the diluted cDNA were then performed using the routine approach (see Supplementary material **B** and **C**).

Spiking experiments

For exact counting of LNCaP cells, we used the FACSAria IIu (BD Biosciences, Franklin Lakes, USA) FACS sorter. Five-hundred thousand trypsinized LNCaP cells

were diluted in 1ml RPMI1640, further separated using a 35µm filter and sorted with electronic gates set on live cells by a combination of forward and side scatter and Sytox Blue exclusion (Life Technologies, Carlsbad, USA). Based on forward and side scatter area versus width parameters, we were able to systematically exclude doublets. We transferred 5, 50 and 500 cells into fresh blood samples of a healthy male (PSA value 0.3ng/ml) and female donor and stored the spiked blood in EDTA collection tubes. Blood samples without LNCaP cells served as negative control. Samples were then subjected to the standard analysis procedure, including AdnaSelect CTC isolation, qPCR and subsequent mutation analysis by qPCR pyrosequencing using the routine approach (see Supplementary material **B** and **C**).

B. Quantitative real-time PCR

We carried out PCR reactions under the following cycling conditions with standard ramping rates: 95°C for 5min for initial Hot Start activation, 94°C for 15s, and 60°C for 60s for a total of 50 cycles. With respect to the limit of detection, we performed the experiments in single measurements in order to increase the available amount of cDNA template per reaction and to enhance the detection of the target transcripts. We chose a reaction volume of 20µl including 1.5µl cDNA template per reaction.

C. DNA pyrosequencing

The biotinylated qPCR product (15µl) was immobilized with Streptavidin High performance Sepharose beads (2µl) (GE Healthcare, Solingen, Germany) and PyroMark Binding Buffer (40µl) on a Thermomixer comfort (Eppendorf, Hamburg, Germany) for 5min at 1300rpm (total volume 80µl). We transferred the immobilized single-stranded DNA template into a PyroMark Q24 Plate holder well with PyroMark Annealing buffer (23µl) and sequencing primer (2µl, 200 pmol) using the PyroMark Q24 Vacuum Workstation. Sequencing and analysis was performed according to the manufacturer's instructions (PyroMark Q24 workstation and analysis software, version 2.0, Qiagen, Hilden, Germany).

Supplementary Table 1: Primer and probe sequences

**qPCR Primer**			
**Oligo name**	**Sequence 5' - 3'**		
f_ALAS1	TGATGAACTACTTCCTTGAGAATC		
r_ALAS1	GAATGAGGCTTCAGTTCCA		
f_AR-Ex5	CGACCAGATGGCTGTCATTC		
r_AR-Ex5	ACTTGTGCATGCGGTACTCA		
f_AR-Ex8	GGACTCCGTGCAGCCTATT		
r_AR-Ex8	GGAAAGTCCACGCTCACCA		
f_AR-V7	GGATGACTCTGGGAGAAAAATTC		
r_AR-V7	CTTTCTTCAGGGTCTGGTCATT		
f_G6PD	CCGGGCATGTTCTTCAA		
r_G6PD	AGGGAGCTTCACGTTCTTGTAT		
f_KLK3-PSA	CCCACTGCATCAGGAACAA		
r_KLK3-PSA	TGTGGCTGACCTGAAATACCT		
**Biotinylated qPCR Primer**			
**Oligo name**	**Biotin-TEG**	**Sequence 5' - 3'**	
Bio_f_AR-Ex5	Biotin-TEG	CGACCAGATGGCTGTCATTC	
Bio_r_AR-Ex5	Biotin-TEG	ACTTGTGCATGCGGTACTCA	
Bio_f_AR-Ex8	Biotin-TEG	GGACTCCGTGCAGCCTATT	
Bio_r_AR-Ex8	Biotin-TEG	GGAAAGTCCACGCTCACCA	
**Sequencing Primer**			
**Oligo name**	**Sequence 5' - 3'**		
seq_f_AR-Ex5	GATGGCTGTCATTCAGTACTCC		
seq_r_AR-Ex5	ACCAGATCAGGGGCGAA		
seq_f_AR-Ex8	TCCGTGCAGCCTATTGC		
seq_r_AR-Ex8	CGCTCACCATGTGTGACTTG		
**TaqMan MGB Probes**			
**Probe name**	**Dye**	**Sequence 5' - 3'**	**Quencher**
p_ALAS1	VIC	CTAGTCACATGGAAGCAA	MGB-NFQ
p_AR-Ex5	6FAM	CTTCGCCCCTGATC	MGB-NFQ
p_AR-Ex8	6FAM	CCTGCTAATCAAGTCACAC	MGB-NFQ
p_AR-V7	6FAM	CAATTGCAAGCATCTCA	MGB-NFQ
p_G6PD	VIC	TCCAGCTCCGACTCC	MGB-NFQ
p_KLK3-PSA	6FAM	CTGTTTCATCCTGAAGACA	MGB-NFQ

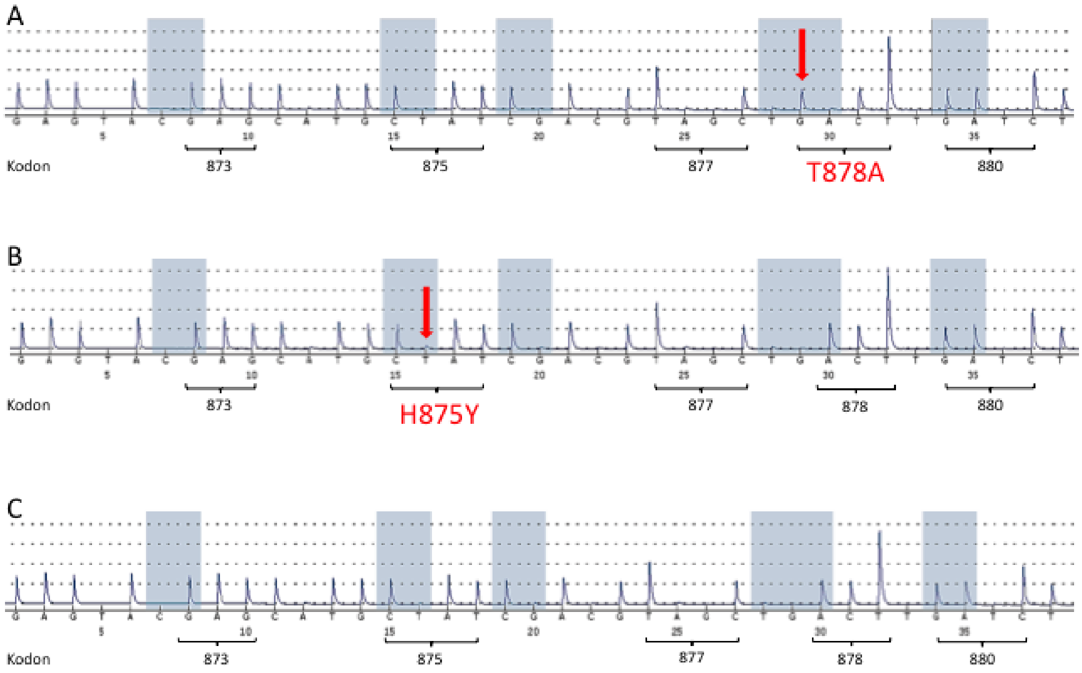

Supplementary Figure 1: cDNA pyrosequencing of AR hotspots.

A, detection of a T878A point mutation (patient no. 03; mutant-to-wildtype ratio, 0.92). **B,** detection of a H875Y point mutation (patient no. 29; allelic ratio, 0.11). **C,** cDNA pyrosequencing of wild-type AR.

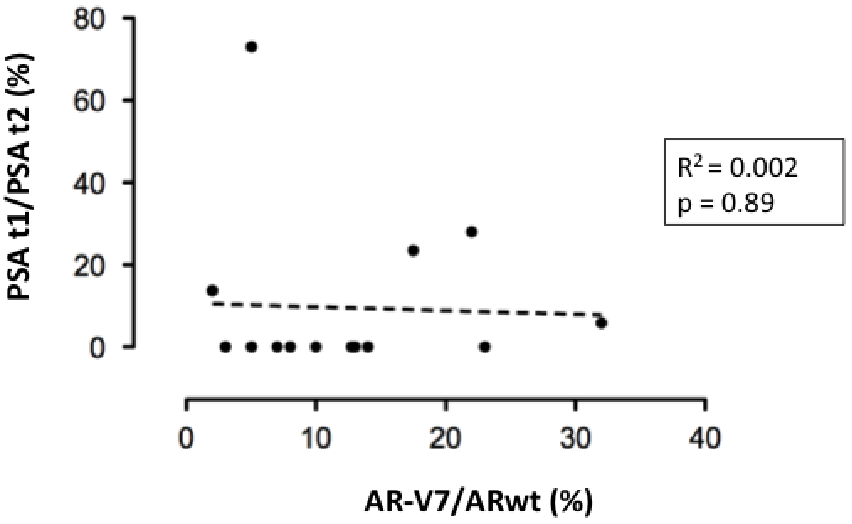

Supplementary Figure 2: Correlation of AR-V7/ARwt with max. PSA-response

Figure legend: PSA t1, prostate specific antigen level before therapy switch; PSA t2, prostate specific antigen nadir after therapy switch; AR-V7, androgen receptor (splice variant 7); ARwt, androgen receptor (wild-type).
